# Enriched environment reduces glioma growth through immune and non-immune mechanisms in mice

**DOI:** 10.1038/ncomms7623

**Published:** 2015-03-30

**Authors:** Stefano Garofalo, Giuseppina D’Alessandro, Giuseppina Chece, Frederic Brau, Laura Maggi, Alessandro Rosa, Alessandra Porzia, Fabrizio Mainiero, Vincenzo Esposito, Clotilde Lauro, Giorgia Benigni, Giovanni Bernardini, Angela Santoni, Cristina Limatola

**Affiliations:** 1 Department of Physiology and Pharmacology, Istituto Pasteur-Fondazione Cenci Bolognetti Sapienza University, Piazzale Aldo Moro 5, 00185 Rome, Italy; 2 Université Nice-Sophia Antipolis, IPMC CNRS-UMR, 7275 Valbonne, France; 3 Department of Biology and Biotechnology Charles Darwin, Sapienza University, Piazzale Aldo Moro 5, 00185 Rome, Italy; 4 Department of Molecular Medicine, Istituto Pasteur-Fondazione Cenci Bolognetti, Sapienza University, Piazzale Aldo Moro 5, 00185 Rome, Italy; 5 Department of Experimental Medicine, Sapienza University, Piazzale Aldo Moro 5, 00185 Rome, Italy; 6 IRCCS Neuromed, Via Atinense 18, 86077 Pozzilli, IS, Italy; 7 Department of Neurology and Psychiatry, Sapienza University, Piazzale Aldo Moro 5, 00185 Rome, Italy

## Abstract

Mice exposed to standard (SE) or enriched environment (EE) were transplanted with murine or human glioma cells and differences in tumour development were evaluated. We report that EE exposure affects: (i) tumour size, increasing mice survival; (ii) glioma establishment, proliferation and invasion; (iii) microglia/macrophage (M/Mφ) activation; (iv) natural killer (NK) cell infiltration and activation; and (v) cerebral levels of IL-15 and BDNF. Direct infusion of IL-15 or BDNF in the brain of mice transplanted with glioma significantly reduces tumour growth. We demonstrate that brain infusion of IL-15 increases the frequency of NK cell infiltrating the tumour and that NK cell depletion reduces the efficacy of EE and IL-15 on tumour size and of EE on mice survival. BDNF infusion reduces M/Mφ infiltration and CD68 immunoreactivity in tumour mass and reduces glioma migration inhibiting the small G protein RhoA through the truncated TrkB.T1 receptor. These results suggest alternative approaches for glioma treatment.

## Abstract (web summary)

Enriched environment is known to be beneficial in several disease settings. Here the authors show that mice pre-exposed to enriched environment survive longer when challenged with glioma due to increased antitumour immunity, and identify soluble factors that mediate these effects.

## Graphical abstract

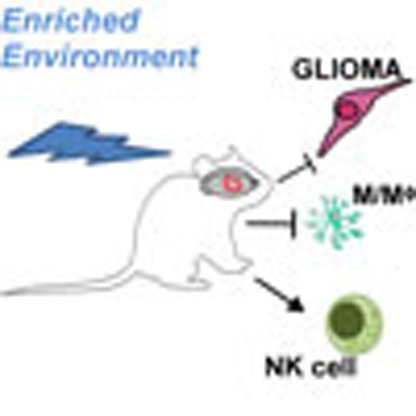

Exposure to an enriched environment (EE), obtained with prolonged physical, social and sensory stimulation, has been described as beneficial for better recovery from several neuropathologies such as stroke, Parkinson’s disease, epilepsy and Alzheimer’s disease^1^. In animal models, EE exposure increases hippocampal neurogenesis, improves performances in several memory tests, induces anatomical and functional changes in synaptic connections, increasing dendrite spine density and potentiating long-term synaptic transmission and modulates cytokine and growth factor levels both centrally, in the cerebral liquor and peripherally, in the plasma^2^,^3^.

Less clear is the effect of EE on cancer, despite the relevance of the issue. Significant reduction of growth, incidence and proliferation of subcutaneous implanted melanoma and colon cancer is reported on EE housing, with involvement of hypothalamic brain-derived growth factor (BDNF), sympathetic innervation of white fat and inhibition of leptin levels^4^. However, less unambiguous is the effect of EE on B-cell lymphoma or breast cancer, and discordant results on the effect on tumour growth in mice housed in EE compared with those housed in standard environment (SE) are reported from different laboratories, with possible variations because of differences in experimental conditions and other unchecked parameters, such as microbiota^5^,^6^,^7^. Of note, EE has important effects on immunological parameters, as well as on the activation of immune effector cells that play relevant roles in the control of transformed cells, including natural killer (NK) cells^8^.

Here we investigated for the first time the effect of EE on progression and development of glioma in mice. We choose malignant glioma as it is the most diffuse and aggressive neoplasm of the nervous system, characterized by high invasiveness and proliferation, diffuse apoptosis and/or necrosis, astrogliosis and microglia/macrophage (M/Mφ) activation, with a poor prognosis. To this aim, we transplanted murine glioma (GL261) into the brain of mice housed in EE or in SE. Results show that EE exposure reduces tumour size and proliferation rate of glioma cells, with increased survival. Similar results on tumour size are also obtained with the human U87MG and with the stem-like CD133^+^ GL261 glioma cells. Interleukin-15 (IL-15) and BDNF are two key mediators of these effects, since they increase in the brain of EE mice, and their *in situ* administration reduces tumour size in glioma-bearing mice, similarly to EE. We describe that IL-15 increases NK1.1^+^ and CD69^+^ cell infiltration in tumour area and that NK cell depletion reduces the efficacy of IL-15 administration on tumour growth and of EE on tumour growth and mice survival. BDNF has direct effects on tumour cells, reducing glioma cell migration *in vitro* and impairing the expression of the phagocytic marker CD68 on M/Mφ, and their infiltration in the tumour mass. These novel results encourage clinical trials testing the effect of EE, IL-15 and BDNF in humans suffering from malignant glioma.

## Results

### Enriched environment affects glioma growth and mice survival

At weaning (21 day old), *C57BL/6* mice were housed for 5 weeks in SE or EE, as described in Methods (Supplementary Fig. 1a). As control of EE efficacy, the effect on hippocampal synaptic plasticity was tested^9^: mice housed in EE (hereafter EE mice) have a robust increase in long-term potentiation (LTP) expression, versus SE mice, as measured by field excitatory postsynaptic potential (fEPSP) recordings in the CA1 region (Supplementary Fig. 1b; LTP evoked by 100 Hz train). EE mice also have a 7% reduction of body weight compared with SE mice (Supplementary Fig. 1c), likely because of the increased physical activity.

**Figure 1:**
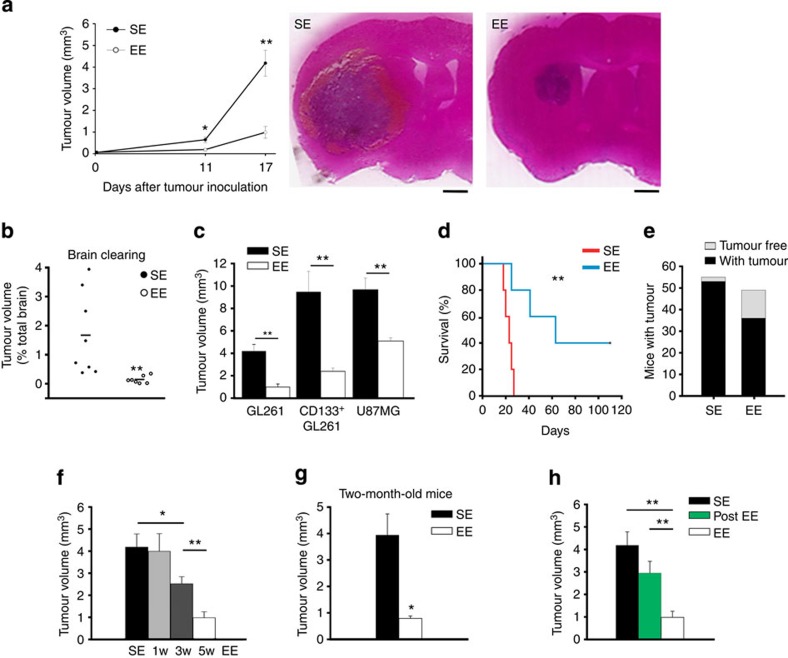
Exposure to EE reduces glioma growth and prolongs survival. (**a**) The mean tumour volumes at 11 and 17 days after implantation of glioma cells into the striatum of C57BL/6 mice, housed in SE or EE, as indicated, from at least 11 mice per conditions. *P<0.05, **P<0.01, Student’s *t*-test. Representative coronal brain sections are shown on the right for each condition at 17 days. Scale bar, 1 mm. (**b**) Quantification of tumour volumes under the same experimental conditions as in **a**, at 17 days, with the brain-clearing technique (*n*=7–8; **P<0.001 Student’s *t*-test). Representative Movies are shown as Supplementary Material.(**c**) The mean tumour volumes (±s.e.m.) at 17 days after implantation of GL261, purified CD133^+^ GL261 or U87MG glioma cells into the striatum of *C57BL/6*, or *SCID* mice housed in SE or EE, as indicated; seven animals per condition. **P<0.01, Student’s *t*-test. (**d**) Kaplan–Meier survival curves of SE (red) and EE (blue) GL261 glioma-bearing mice (*n*=5 per group; log-rank test **P<0.01). (**e**) Tumour resistance in SE and EE mice expressed as total number of mice with (black) and without (grey) brain tumour 17 days after GL261 transplantation. (**f**) ‘Dose dependency’ of EE exposure on tumour volume (*n*=7 per group; *P<0.05; **P<0.01, one-way ANOVA. (**g**) Effect of SE or EE housing on 2-month-old mice transplanted with glioma (*n*=5 per group; *P<0.05, Student’s *t*-test). (**h**) Effect of housing in SE and EE before (EE, for 5 weeks) or after tumour transplantation (post-EE, for 17 days) on tumour volume (all mice analysed 17 days after tumour transplantation). Data are from at least five mice per group. **P<0.01, Student’s *t*-test.

After this period, *C57BL/6* mice were brain-transplanted with syngeneic GL261 glioma cells (7.5 × 10^5^ cells) in the right striatum, left in their original cages and analysed at different times for tumour volume. Figure 1a shows that 11 days after glioma transplantation, the brains of EE mice have smaller tumours and the difference is maintained at 17 days with a reduction in tumour volume to 23.9±6% of SE. Tumour size (from now on analysed on 17 days) was also evaluated with the brain-clearing technique and ultramicroscopy (using tag Red Fluorescent Protein (RFP) GL261, see Methods), confirming an unambiguous reduction in the brain of EE mice (Fig. 1b and Supplementary Movies 1–4). The reduction in tumour size was also observed with different glioma cells, namely the human U87MG and the stem-like CD133^+^ murine GL261 cells (Fig. 1c).

Enriched housing significantly has an impact on the survival time of glioma-bearing mice (Fig. 1d): SE mice have a mean survival time of 22.6±1.6 days that increases to 65.8±15.2 days in EE mice. Interestingly, EE mice also have an increased resistance to develop the injected tumour: in SE, 2/55 injected mice (3.5%) do not develop the transplanted tumour, while in EE mice this value raises to 13/49 (26.5%, Fig. 1e).

Additional experiments were performed to evaluate the time dependency of EE exposure. With this aim, mice were exposed to EE for 1, 3 or 5 weeks before tumour transplantation, and analysed as above, after 17 days. Results shown in Fig. 1f demonstrate that 1 week is ineffective, while 3 weeks of EE exposure are sufficient to obtain significant reduction in glioma size, suggesting a possible ‘dose-dependent’ effect. We also investigated whether the age of mice at the beginning of the EE protocol could influence the effects on glioma growth. For this reason 2-month-old mice (adult) were housed in EE or SE and transplanted with GL261 as described. Data reported in Fig. 1g demonstrate that exposure to EE has similar effects on adult in comparison with weaned mice (Fig. 1a), thus excluding an age-dependent effect, at least for the periods taken into account. We also evaluated whether exposure to EE simultaneously to glioma transplantation could be efficient in contrasting the tumour growth: results reported in Fig. 1h indicate, after 17 days, a non- significant tendency to reduction between this group of mice (post-EE) and those housed in SE, in contrast to EE mice (**P<0.01, Student’s *t*-test, post-EE versus EE group). These data indicate that the effect of EE housing is significant when the brain is conditioned before tumour implantation and likely requires longer periods to become effective.

**Figure 2:**
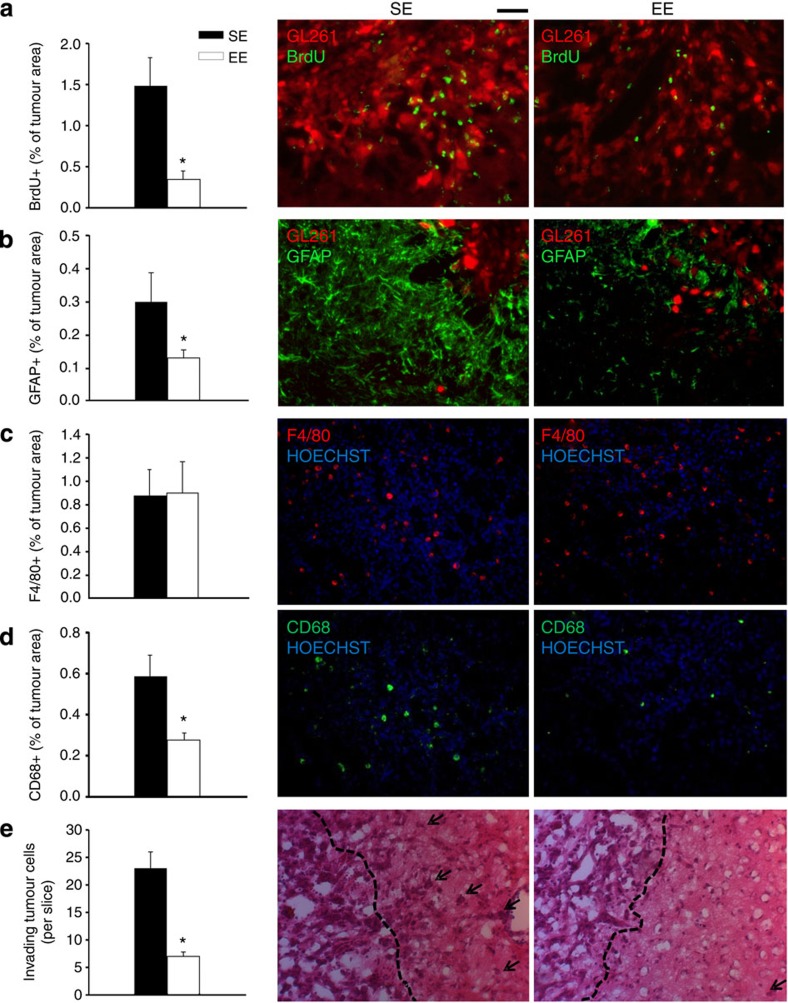
EE modulates tumour microenvironment. (**a**) The mean (±s.e.m.) area of BrdU^+^ cells in tumour area (as % of the tumour area) at 17 days after tagRFP-GL261 implantation in mice housed in SE or EE, as indicated (*P<0.05; Student’s *t*-test; *n*=4 mice per condition). Representative immunofluorescence of proliferating BrdU^+^ cells (green) under the two experimental conditions are shown on the right. Red: tagRFP-GL261 cells. (**b**) The mean (±s.e.m.) area of GFAP^+^ cells (as % of the tumour area, *P<0.05, Student’s *t*-test, *n*=4 mice per condition) 17 days after tagRFP-GL261 transplantation in mice housed in SE or EE, as indicated. Representative immunofluorescences of astrogliosis, seen as GFAP^+^ cells (green) are shown on the right for the two experimental conditions. Red: tagRFP-GL261 cells. (**c**,**d**) The mean (±s.e.m.) area of F4/80^+^ (**c**) and CD68^+^ (**d**) cells (as % of the tumour area, *n*=3–4 mice per condition) 17 days after GL261 transplantation in mice housed in SE or EE, as indicated. Right: representative immunofluorescences of M/Mφ glioma infiltration (**c**, F4/80 in red) and activation (**d**, CD68 in green, *P<0.05 Student’s *t*-test) 17 days after GL261 transplantation. Nuclei are evidenced with Hoechst (blu). (**e**) The mean number (±s.e.m.) of glioma cells invading the brain parenchyma for more than 150 μm beyond tumour border 17 days after glioma cell transplantation in SE or EE (*n*=4 mice per condition; *P<0.05 Student’s *t*-test). Right: representative coronal brain sections stained with haematoxylin/eosin. Black arrows indicate glioma cells invading the brain parenchyma beyond the main tumour border (dashed line) for more than 150 μm. For all the panels: scale bars, 10 μm.

The brains of SE or EE mice were compared for cell proliferation in tumour mass (as 5-bromo-2′-deoxyuridine (BrdU) incorporation), astrocyte activation at the tumour border (glial fibrillary acidic protein, GFAP^+^), M/Mφ infiltration (F4/80^+^) and activation of phagocytic activity (CD68^+^) within the tumour. Data shown in Fig. 2a show that EE affects cell proliferation, reducing the extent of BrdU^+^ cells in tumour mass. Astrogliosis is also reduced by EE (Fig. 2b), while M/Mφ infiltration is not affected (Fig. 2c). In contrast, CD68^+^ cells are significantly reduced in EE in comparison with SE and are only detected in the tumour region, and not in healthy parenchyma (Fig. 2d). CD68 staining is confined to M/Mφ population (as verified by co-labelling with GFP^+^ cells in *CX3CR1*^*+/GFP*^ mice, Supplementary Fig. 2), being completely absent in glioma cells.

Cerebral slices were also analysed for glioma cell invasiveness towards the healthy brain parenchyma in SE and EE conditions. We observe that EE mice have a reduced number of glioma cells protruding more than 150 μm from the main tumour mass (Fig. 2e), indicative of a reduced tendency to migrate and invade the surrounding tissue.

### Enriched environment changes the glioma microenvironment

In order to identify the factors responsible for the beneficial effects of EE in the brain of glioma-bearing mice, real-time–PCR (RT–PCR) analyses were performed on cerebral hemispheres of SE and EE mice, on sham insult or glioma transplantation. On EE exposure, a significant increase in BDNF is observed in both the hemispheres of sham-operated (Supplementary Table 1; *P*<0.05, Student’s *t*-test, *n*=3) and glioma-bearing mice (Table 1; *P*<0.01, Student’s *t*-test, *n*=4). Comparable results are obtained measuring BDNF protein levels using enzyme-linked immunosorbent assay (ELISA; see Supplementary Fig. 3). Differently, IL-15 mRNA levels increase, on EE, in both brain hemispheres of tumour-bearing mice (Table 1), not increasing in sham-operated mice (Supplementary Table 1). Other chemokines and cytokines known to be involved in glioma progression, such as CCL2, CXCL10 and IL-6, are selectively upregulated in the glioma-bearing hemisphere, and these increases are strongly reduced in EE mice (Table 1).

We then attempted to identify the source of IL-15 and BDNF produced in the brain of EE mice, and we focused on CD11b^+^ cells, mainly comprising microglia, macrophages, NK and dendritic cells. To this aim, highly purified (>90%) CD11b^+^ cells were isolated from the brain of SE and EE glioma-bearing mice and analysed for cytokine expression (using RT–PCR). Data shown in Table 2 indicate that, in EE mice, IL-15 increase is restricted to CD11b^+^ cells, while similar increase in BDNF levels is observed in CD11b^+^ and CD11b^−^ cells.

### IL-15 promotes NK cell accumulation in the tumour site

**Figure 3:**
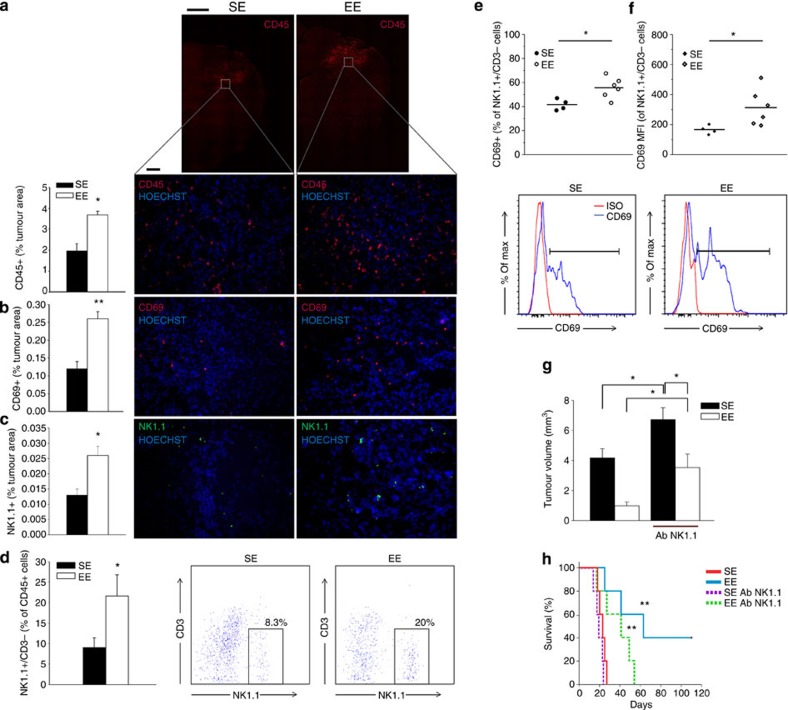
EE increases NK cell infiltration in the tumour area. (**a**) CD45^+^ cells increased in the tumour area of EE-housed mice (*n*=4; *P<0.05 Student’s *t*-test). Representative immunofluorescence is shown on the right, at low (× 4, top, scale bar 1 mm) and high (× 20, bottom, scale bar 0.1 mm also for **b** and **c**) magnification. (**b**) CD69^+^ cells increased in the tumour area of EE-housed mice (*n*=4; **P<0.01 Student’s *t*-test). Representative immunofluorescence is shown on the right. (**c**) Analysis of NK1.1 cell infiltration in tumour, expressed as % NK1.1^+^ cells in the tumour area (±s.e.m.; *P<0.05 Student’s *t*-test; *n*=3–4 mice per condition). Representative immunofluorescence of NK1.1^+^ cells (green) 17 days after glioma-cell transplantation in mice housed in SE or EE are shown on the right. (**d**) CD3^−^/NK1.1^+^cells increased in the brain hemisphere injected with glioma on housing in EE (*n*=6; *P<0.05 Student’s *t*-test). Representative FACS analysis of CD3^−^/NK1.1^+^ cells is shown on the right. (**e**) Percentage of CD69^+^ cells in the CD3^−^/NK1.1^+^ cell population obtained from the brain of SE or EE mice (*n*=6; *P<0.05, Student’s *t*-test). Representative FACS analyses are shown below. (**f**) Mean fluorescence intensity (MFI) of CD69 staining in CD3^−^/NK1.1^+^ cell population obtained from the brain of SE or EE mice (*n*=6; *P<0.05, Student’s *t*-test). (**g**) Analysis of tumour volume (expressed as mm^3^±s.e.m.) in vehicle and NK1.1 Ab-treated mice, housed in SE or EE; *n*=5; *P<0.05, two-way ANOVA). (**h**) Kaplan–Meier curve of glioma-transplanted mice exposed to SE or EE on NK1.1 Ab treatment; *n*=5; log-rank test **P<0.01.

We focused our attention on the role played by IL-15 in the brain of EE glioma-bearing mice. Since it is known that IL-15 regulates NK cell proliferation/maturation and effector functions, we wondered whether EE resulted in an increased number and activation state of NK cell-infiltrating brain parenchyma. Preliminarily, we analysed the brain of SE and EE mice for leukocyte infiltration and activation by the expression, respectively, of CD45^+^ and CD69^+^ markers and data shown in Fig. 3a,b indicate that these cells, confined in the tumour area, increase in EE. Sham-injected mice have no brain-infiltrating CD45^+^ cells, independently of housing conditions (0.14±0.06% of total, *n*=6). Similar evidence of cell infiltration was obtained using haematoxylin/eosin staining (Supplementary Fig. 4a). Brains of EE mice also have a significant increase in NK1.1^+^ cells, as evidenced by staining slices with anti-NK1.1 monoclonal antibody, that recognizes an epitope of the NKR1Pc-activating receptor (Fig. 3c). Leukocytes were also isolated from the brain by Percoll gradient and analysed using immunofluorescence and fluorescence-activated cell sorting (FACS): data shown in Fig. 3d confirm, among the infiltrated leukocytes, an increased frequency of NK cells (specifically in the ipsilateral hemisphere, evidenced as NK1.1^+^/CD3^−^/CD45^+^) in EE versus SE mice. No significant differences in NKT cell (NK1.1^+^/CD3^+^/CD45^+^) frequency is observed in the brain of SE and EE mice (SE: 0.22±0.07%; EE: 0.38±0.03%). Interestingly, NK1.1/CD3/CD69 co-staining demonstrates that the CD69^+^ cell population modulated by EE is mainly composed of NK cells (Fig. 3e), being CD69 not increased in T-cell population (NK1.1^−^/CD3^+^/CD69^+^ cells, SE: 7.65±2.59%; EE: 9.20±2.01%, *n*=6). NK cell population also has increased CD69 mean fluorescence intensity on EE (Fig. 3f), indicating increased activation state. The functional state of these cells is also confirmed by granzyme B (9.76±3.2%) and interferon (IFN)-γ (5.19±1.7%) expression (*n*=6). All together, these data demonstrate an increase in activated antitumour effector cells in the brain of EE mice. Higher frequency of NK cells is also observed in the blood of EE mice, with an increased capacity to produce IFN-γ in response to cytokine stimulation (Supplementary Fig. 4b). Accordingly, in the brain of EE mice we observe the increased expression of another NK cell activating cytokine, IL-2, with no variation in IL-18, IL-12p35 or IL-12p40 (Supplementary Table 2).

To further analyse the role played by NK cells, we decided to selectively deplete them in glioma-bearing mice by repeated injections of anti-NK1.1 Ab (performed at −2, 0,+2 and +7 days from glioma injection). The evaluation of the frequency of NK cells (CD3^−^/NK1.1^+^) at +7 and +17 days in the peripheral blood confirms successful NK cell depletion in both SE and EE mice (Supplementary Fig. 4c). NK cell-depleted mice always have larger tumours, in comparison with non-depleted ones (Fig. 3g), demonstrating that glioma is susceptible to the antitumour activity of NK cells. Interestingly, the effects of EE on tumour size are strongly reduced but not abolished in NK cell-depleted mice (47% reduction) in comparison with normal mice (75% reduction; *P*<0.05, two-way analysis of variance (ANOVA)). The effect of NK cell depletion was also investigated on mice survival: data in Fig. 3h show that NK cell-depleted mice survive significantly less when exposed to EE (37.6±6.9 versus 65.8±15.2 days in EE-undepleted mice, *P*<0.01, log-rank test). Similarly to what described for tumour size, the effect of EE is reduced but maintained on NK cell depletion (SE depleted: 20.2±2 versus EE depleted: 37.6±6.9 days, *P*<0.01 log-rank test). No significant difference in survival time is observed in mice housed in SE, upon NK cell depletion. All together, these data demonstrate that EE affects tumour size and mice survival through both NK cell-dependent and NK cell-independent mechanisms.

**Figure 4:**
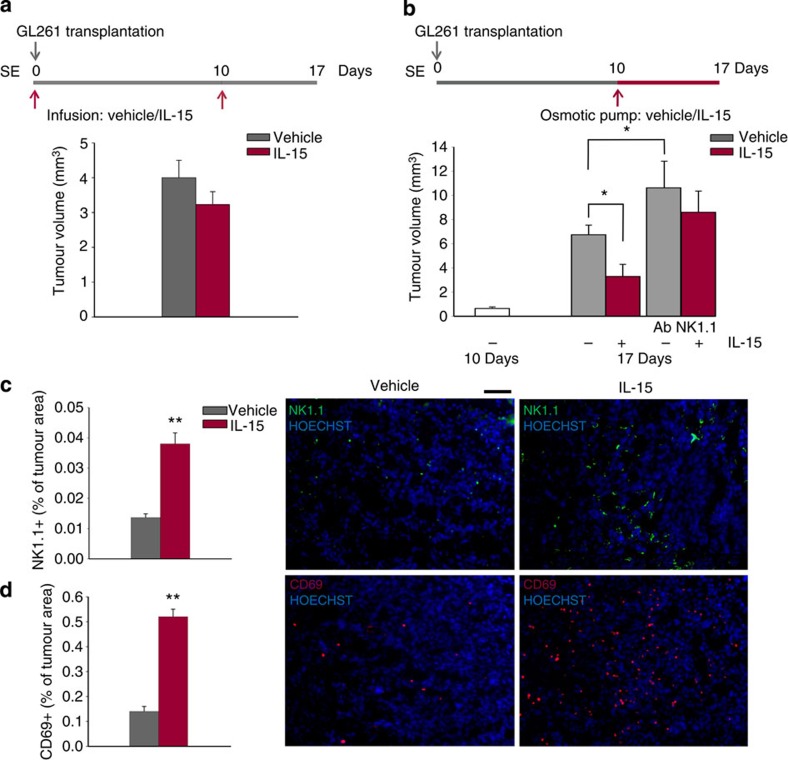
IL-15 administration impairs glioma growth and increases NK cell infiltration. (**a**) Effects on tumour volume of IL-15 or vehicle infusion into the striatal region of the glioma-injected hemisphere in SE mice, administrated as described in the scheme on top. Brains were analysed 17 days after glioma transplantation. Graph bars illustrate the mean tumour volume (±s.e.m.; *n*=4 mice per condition). (**b**) Effects of intrastriatal infusion of vehicle or IL-15 with micro-osmotic pumps starting 10 days after glioma cell transplantation and for 7 days, in SE mice, as described on top (mean tumour volume±s.e.m.; *P<0.05 Student’s *t*-test; *n*=4–6 mice per condition). (**c**,**d**) NK cell infiltration (**c**, NK1.1) and activation (**d**, CD69) in the glioma of mice treated with vehicle or IL-15, with the protocol shown in **b**. Graph bars represent the mean area (±s.e.m.) as percentage of total tumour area. Representative immunofluorescences are shown on the right (scale bar, 100μm. **P<0.01 Student’s *t*-test; *n*=3–4 mice per condition).

We then investigated whether a protective effect could be also obtained by exogenous IL-15 administration. To this aim, SE mice were brain-injected with vehicle or IL-15 (50 ng ml^−1^, 3 μl) on day 0 and 10 after glioma transplantation and brains were analysed at day 17. Results shown in Fig. 4a demonstrate that this treatment is ineffective in reducing tumour size. In contrast, when IL-15 is continuously delivered to SE mice with micro-osmotic pumps from day 10 to 17 after glioma transplantation (50 ng ml^−1^, 100 μl, 0.5 μl h^−1^), a significant reduction in tumour size is observed (Fig. 4b). In these mice, brain infiltration of NK (that is, NK1.1^+^/CD3^−^/CD45^+^) cells is significantly increased (Fig. 4c). Interestingly, IL-15 infusion with micro-osmotic pumps also strongly increases the total number of activated CD69^+^ cells (Fig. 4d). When IL-15 is infused in the brain of NK cell-depleted mice, no significant reduction in tumour volume is observed, demonstrating that NK cells are necessary for the antitumour activity of IL-15 (Fig. 4b).

### BDNF reduces tumour size and M/Mφ infiltration

**Figure 5:**
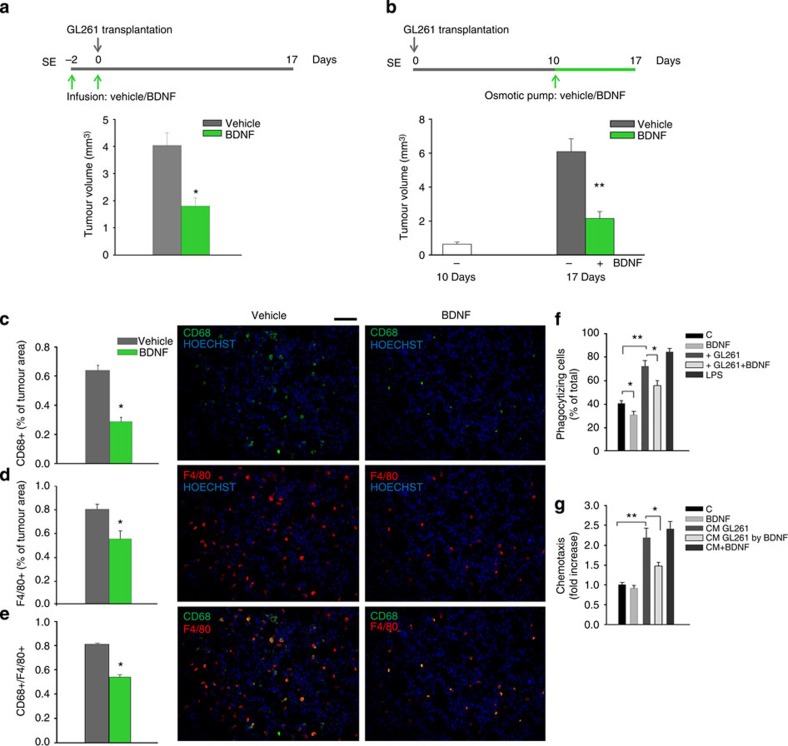
BDNF administration impairs glioma growth and reduces M/Mφ infiltration and activation. (**a**) Effects of BDNF (or vehicle) infusion into the ipsilateral striatum on tumour volume of SE mice, administrated as shown in the scheme on top. Data are shown as tumour volume (in mm^3^)±s.e.m.; *P<0.05, Student’s *t*-test; *n*=4 mice per condition. (**b**) Effects of intrastriatal infusion of vehicle or BDNF with micro-osmotic pumps starting 10 days after glioma cell transplantation and for 7 days, in SE mice, as described on top. Graph bars illustrate the mean tumour volume in mm^3^ (±s.e.m.; **P<0.01 Student’s *t*-test; *n*=4–6 mice per condition). (**c**–**e**) M/Mφ activation (**c**, CD68) and infiltration (**d**, F4/80) in the glioma of mice treated with BDNF or vehicle, as shown in **b**, at the end of treatment (17 days from glioma transplantation); (**e**) merged images of F4/80 and CD68 staining. Graph bars represent the mean (±s.e.m.) area as percentage of total tumour area. Representative immunofluorescences are shown on the right (scale bar, 100μm. *P<0.05 Student’s *t*-test; *n*=3–4 mice per condition). (**f**) Phagocytic activity of microglia co-cultured or not for 24 h with GL261 in the presence or in the absence of BDNF (100 ng ml^−1^). LPS (500 ng ml^−1^) was used as positive control. Results are expressed as % of phagocytizing cells, considering positive only cells with more than four FluoSpheres in the cytoplasm (*n*=5; *P<0.05; **P<0.01 one-way ANOVA). (**g**) Microglia chemotaxis towards the control medium (C), BDNF (100 ng ml^−1^) or GL261-conditioned medium (CM) in the presence or absence of BDNF, for 18 h. Control for a direct effect of BDNF/CM was performed by assaying chemotaxis towards GL261 CM supplemented with BDNF only during the chemotaxis assay. Results are expressed as fold increases in comparison with C (*n*=5; *P<0.05, **P<0.01 one-way ANOVA).

To identify the NK cell-independent component of the antitumour activity of EE, we next investigated the potential role played by BDNF on glioma growth: to this aim, SE mice were infused with BDNF (100 ng ml^−1^, 3 μl) or vehicle, 48 h before and during glioma transplantation and their brains were analysed 17 days later. Results shown in Fig. 5a demonstrate that this treatment significantly reduces tumour volume. Similar results are obtained when BDNF (or vehicle) is delivered 10 days after glioma transplantation into the brain of SE mice with micro-osmotic pumps, for 7 days (100 ng ml^−1^, 100 μl, 0.5 μl h^−1^). Figure 5b shows a significant reduction in tumour size in the presence of BDNF, demonstrating an antitumour effect similar to EE. In the brain of mice where BDNF was delivered with micro-osmotic pumps, we also observe a significant reduction of CD68^+^ cells (Fig. 5c), indicative of a reduced phagocytic activity of M/Mφ. Interestingly, under these conditions, also the total number of F4/80^+^ cells is reduced (Fig. 5d), demonstrating a BDNF-dependent reduction in M/Mφ infiltration of the tumour mass. All CD68^+^ cells are M/Mφ, as verified by CD68/F4/80 co-staining (Fig. 5e).

We then tested, *in vitro*, the effect of BDNF on microglia functions. Data shown in Fig. 5f,g indicate that BDNF is ineffective in directly modulating either phagocytic or chemotactic activity of the microglia. However, BDNF hampers the increase in phagocytic (Fig. 5f) and chemotactic (Fig. 5g) activities induced on the microglia by co-culturing with glioma cells (Fig. 5f,g). All together, these data suggest that BDNF induces the release of soluble factors by the glioma, acting on microglia to modulate phagocytosis and chemotaxis.

**Figure 6:**
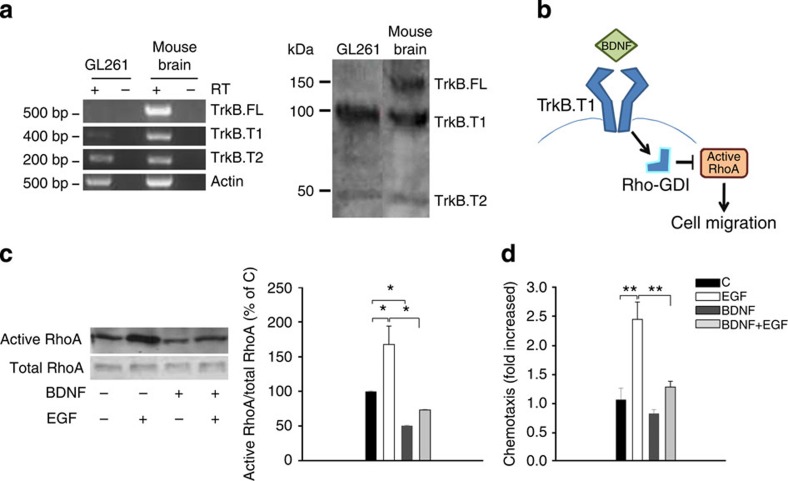
BDNF impairs glioma cell motility *in vitro* by the activation of TrkB-T1. (**a**) RT–PCR (left) and western blot (right) analyses of TrkB isoform expression in GL261 and the mouse brain. (**b**) Proposed model to illustrate TrkB.T1 signalling on BDNF binding, impairing RhoA activation and cell migration. (**c**) Analysis of active RhoA in GL261 glioma cells on BDNF (100 ng ml^−1^) and/or EGF (100 ng ml^−1^) stimulation (5 min). Representative experiment is shown on the left. Data were normalized to total RhoA and expressed as percentage of untreated cells (C; *n*=4; *P<0.05 one-way ANOVA). (**d**) GL261 cell chemotaxis (18 h) towards BDNF (100 ng ml^−1^) and/or EGF (100 ng ml^−1^). Chemotaxis is expressed as the fold increase in comparison with C (*n*=5; **P<0.01 one-way ANOVA).

To further investigate the direct effects of BDNF on glioma, we analysed the expression of TrkB receptors on GL261 cells using RT–PCR and western blot analyses. Data shown in Fig. 6a demonstrate that GL261 cells do not express the full-length form of TrkB (TrkB.FL) but do express the truncated forms TrkB.T1 and TrkB.T2, both as mRNA (left) and protein (right). The TrkB.T1 receptor signals to Rho protein dissociation inhibitor (RhoGDI) that, on BDNF stimulation, detaches from TrkB.T1 and binds to the small G protein RhoA, leading to its inhibition^10^ (see scheme in Fig. 6b). Data shown in Fig. 6c show that the BDNF treatment of GL261 cells reduces the basal activation state of RhoA and completely abolishes epidermal growth factor (EGF)-induced RhoA activation, as evaluated by a Rhotekin-GST pull-down assay. Other signalling components activated by EGF, including FAK and ERK1/2, are inhibited by BDNF co-treatment (Supplementary Fig. 5a). Functional analysis of cell migration correlates with BDNF signalling: Fig. 6d shows that GL261 cells are not attracted by BDNF but migrate towards EGF and, similarly to the effects on signalling, EGF-induced GL261 migration is completely abolished by BDNF co-treatment.

BDNF has no direct effect on the GL261 cell proliferation rate and, at variance with data on migration and signalling, the proliferative activity of EGF is not hampered by BDNF co-treatment (Supplementary Fig. 5b).

## Discussion

**Figure 7:**
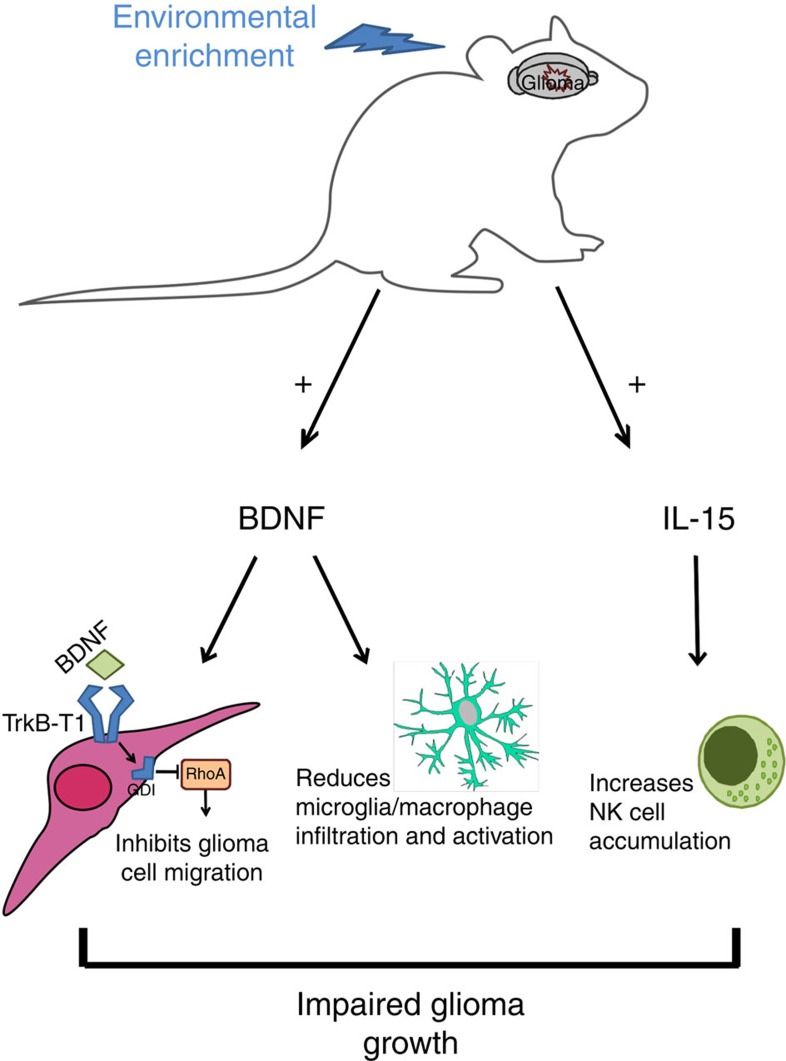
Model of EE-induced mechanisms impairing glioma growth through BDNF and IL-15. Housing in EE induced brain increase of IL-15 and BDNF. IL-15 modulates NK cell accumulation and activation in the brain. BDNF has direct effects on the glioma, impairing chemotaxis, and indirect effects on M/Mφ, reducing cell infiltration in the brain parenchyma and phagocytic activity. All these effects contribute to reducing glioma growth in the mouse brain. See discussion for details.

In this paper we report for the first time that housing in EE negatively modulates the growth of mouse and human intracranial glioma and significantly prolongs mice survival. We demonstrate that the reduction in glioma size in EE-housed mice is mediated by an increase in brain IL-15, which favours NK cell accumulation and antitumour function, and BDNF, which suppresses M/Mφ infiltration and activity, and directly affects tumour cell migration (see Fig. 7).

Housing in EE is known to modify the brain microenvironment, with beneficial effects on cognitive processes, increase in neural plasticity and direct influence on neurogenesis, dendritic spine density and synaptic protein synthesis^9^,^11^. Enriched social, sensory and physical activities are reported to partially prevent the onset and to reduce the devastating impact of several neurodegenerative diseases in animal models and in humans^12^,^13^, a phenomenon often referred to as brain cognitive reserve^14^,^15^. The progression of malignant glioma is directly related to its capability to produce soluble mediators causing neuron killing, degradation of extracellular matrix and recruitment of local and peripheral cells, all events contributing to tumour expansion. For all these reasons, we wondered whether EE could interfere with the ability of glioma to create a microenvironment favourable to its expansion. Our results show that housing in EE has antitumour activity, reducing glioma growth in the brain, as assessed by classic staining methods and with the brain-clearing technique^16^ by using RFP-tagged GL261 cells. The inhibitory effect of EE is already evident at 11 days after tumour transplantation, persists until day 17 and parallels the reduction of cell proliferation (BrdU staining) in the tumour mass. Likely as a direct consequence of the reduced tumour expansion, housing in EE significantly prolongs the survival time of glioma-bearing mice and, intriguingly, also reduces the incidence of tumour development on glioma transplantation. The effect of EE on tumour size is not limited to a single cell line, being also obtained with the human U87MG xenografted in severe combined immunodeficient (SCID) mice and with a purified CD133^+^ GL261 cell population.

The effect of experience in EE on tumour growth was investigated in a syngeneic murine model of mammary carcinoma, where EE reduced tumour growth, at least in the initial phase^5^. On EE housing, mice with subcutaneous implanted melanoma or colon cancer had reduced tumour development and progression, with mechanisms involving hypothalamic BDNF and sympathetic modulation^4^. However, these results were not successfully reproduced in a different laboratory, encouraging further research with rigorous control of possible confounding variables^7^.

We report that the EE-induced reduction in tumour size is not dependent on the age of the animals at the beginning of the enrichment period, since it is similarly observed when the EE started at three (weaning) or eight (adult) weeks of age. This is in line with other studies showing that the effect of enrichment is not clearly correlated with the age at the onset of differential housing conditions^17^, at variance with environmental (principally sensory) deprivation, where specific critical periods have been identified^18^. On the other hand, we demonstrate that the length of EE exposure is critically important for the efficacy of antitumour activity, suggesting a sort of ‘dose dependency’ of EE on tumour.

We demonstrated that EE mice, in the presence of glioma, have increased mRNA levels of cerebral IL-15, expressed by CD11b^+^ cells. Previous evidence demonstrated that short (3 min) bouts of physical exercise increased the IL-15 plasma level in humans^19^, suggesting that physical exercise modulates the expression of genes involved in the regulation of NK cell maturation, proliferation and activation. However, motor activity is only one of the aspects potentiated in EE and it is not known whether more prolonged exercise, similar to that offered to mice in our experimental protocol, is sufficient to increase cerebral IL-15 in glioma-bearing mice. Interestingly, we never detect increase of IL-15 in EE mice in the absence of glioma, suggesting the requirement of at least two different initiating signals, one from glioma-conditioned microenvironment and one from factor(s) produced in the brain on EE housing.

An attractive hypothesis would be that factors in the brain of EE mice inhibit the acquisition of a pro-tumour phenotype by CD11b^+^ M/Mφ, thus enhancing their capacity to produce IL-15 in response to tumour cells. In this regard, tumour-infiltrating M/Mφ have lower CD68 expression in EE mice as compared to SE mice, suggestive of changes in their phenotype, and this is accompanied by reduced glioma cell proliferation and invasion into surrounding parenchyma, and reduced astrogliosis. Since CD68 is a classic marker of the anti-inflammatory M2-like phenotype^20^, our results would suggest a possible EE-mediated phenotype switch towards a pro-inflammatory, antitumour M1-like phenotype of infiltrating M/Mφ, thus supporting the protective effects. Interestingly, a recent study demonstrated that infusion of NK cells with an Ab against NG2/CSPG4 converted the anti-inflammatory microenvironment of glioblastoma into a pro-inflammatory one, dominated by M1-like M/Mφ^21^.

We demonstrate that IL-15 expression in the tumour-bearing brains of EE mice is associated with *in situ* accumulation of activated CD45^+^/CD69^+^ leukocytes, and specifically of NK cells, that are markedly increased in comparison with SE mice. NK cells play important role in the beneficial effect of EE, since their depletion strongly reduces the effect of the housing condition, both in term of tumour size and survival time. These positive effects are compatible with increased NK cell effector functions against glioma cells, since NK cells isolated from the brain of glioma-bearing EE mice are both granzyme B- and IFN-γ-positive. A similar stimulating effect of EE on NK cell activity and its potential application in tumour immunotherapy has been proposed for B-cell lymphoma^6^,^8^. It remains to be understood whether, in our experimental model, the reduced tumour cell proliferation observed in EE-housed mice (as BrdU staining)is only because of immune-mediated tumour killing.

We show that IL-15 infusion in tumour-bearing SE mice has the same effect of EE both on tumour size and on NK cell accumulation in the tumour area, confirming its potential antiglioma activity^22^. Importantly, we also demonstrate a direct link between IL-15 production and NK cell antitumour activity since IL-15 infusion does not inhibit glioma growth in NK cell-depleted mice. IL-15 can induce NK cell antiglioma activity *in vivo* by promoting their maturation, proliferation and activation *in situ*^23^ or by enhancing NK cell recruitment in the brain^24^,^25^. In addition, recent evidence demonstrated that activated NK cells, in mouse bearing glioma, communicate with M/Mφ to promote the acquisition of a pro-inflammatory, M1-like, antitumour phenotype^21^. All together, these data prompt to consider IL-15 as the possible therapeutic target in glioma.

In this paper we confirm that, in the brain of EE mice, BDNF levels increase^26^,^27^,^28^,^29^. In the brain, BDNF can be produced by different cell types such as neurons^30^,^31^ and microglia^32^, and its increase on EE has been correlated with effects on synaptic plasticity and cognitive functions in mice^33^. We demonstrate that the intracerebral administration of BDNF induces reduction of tumour size and inhibition of M/Mφ infiltration and activation (as CD68 staining). Considering the crucial roles of infiltrating M/Mφ in glioma growth and progression^34^, we hypothesize that the effects of BDNF on M/Mφ, likely induced by cross-talk with glioma, might underlie the observed reduction in tumour size. BDNF has direct effects on the glioma, mediating the functional effects observed on M/Mφ but also reducing the chemotactic activity of tumour cells through activation of TrkB.T1 receptor and inhibition of RhoA signalling, as previously shown^35^. Evidence of neurotrophin receptor signalling in glioma progression is debated: correlative data demonstrate a pro-migratory activity of p75NTR (ref. 36), while more recent studies focused on the growth-promoting activity of p75NTR signalling in glioma stem cells^37^,^38^,^39^. Our data point to an unambiguous inhibitory effect on glioma growth by BDNF signalling.

We observe that other cytokines and chemokines, known to be involved in glioma biology, are modulated in the brain of EE-housed mice, such as IL-6, CCL2 and CXCL10 (ref. 34). These cytokines strongly increase in the glioma-bearing hemisphere of SE mice, and are potently reduced by EE where tumour mass is reduced. However, at the moment, we cannot conclude whether the modulation of expression of these cytokines is the cause or the consequence of tumour volume reduction on EE exposure and whether their reduction affects the quality of leukocyte recruitment towards glioma.

In conclusion, confirming the knowledge that the brain milieu is modulated by environmental stimuli, we now give novel evidence that prolonged sensory, social and physical experiences provide the brain with protective inputs that reduce the probability to develop and favour glioma growth, consistently increasing the survival time of glioma-bearing mice. This environment–brain–tumour connection appears to be mediated by IL-15 and BDNF, cytokines whose level increases in the brain of EE-housed mice. While IL-15 produced by CD11b^+^ cells modulates NK cell infiltration and antitumour activity, BDNF reduces M/Mφ infiltration and the invasion of cerebral parenchyma by glioma cells through Trkb.T1, both contributing to limit glioma expansion. These data open new perspectives for glioma treatment and reinforce the holistic idea that lifestyles, directly having an impact on the brain microenvironment, reduce the progression and help to prevent several brain diseases, among which are tumours.

## Methods

### Materials

Transwell inserts are from BD Labware (Franklin Lakes, NJ); F4/80, Trkb (H-181), FAK (A-17) FAK (Tyr397) Abs are from Santa Cruz Biotechnology (Santa Cruz, CA); GFAP, BrdU Abs are from Novus Biological (Littleton, CO, USA); CD68 Ab are from AbD Serotec (Oxford, UK); phospho ERK1/2 (Thr202/Tyr204), ERK2 Abs, PCR reactions are from New England Biolabs Inc. (Hertfordshire, UK); secondary Abs are from DAKO (Milan, Italy); culture media, B27, fetal bovine serum (FBS), goat serum, penicillin G, streptomycin, glutamine, Napyruvate, recombinant hEGF, red fluorescent FluoSpheres (1 μm diameter), Thermo Script RT–PCR System, Hoechst 33342 are from GIBCO Invitrogen (Carlsbad, CA, USA); BDNF is from Immunological Sciences (Rome, Italy); BDNF E_MAX_ImmunoAssay System is from Promega (Milan, Italy); BrdU, LPS (lypopolisaccharides from Escherichia Coli 0111B4), haematoxylin, eosin, Percoll, tetraHydroFuran, anhydrous (THF), dichloromethane, DiBenzylEther 98% are from Sigma-Aldrich (Milan, Italy); microbeads CD11b+ are from Miltenyi Biotec (Bologna, Italy); RNeasy Mini Kit is from Qiagen (Hilden, Germany); RhoA activation Assay Kit are from Millipore (Merk Millipore, Billerica, MA); CD45, CD69, CD133, NK1.1 Abs, IL-15 are from eBioscience Inc, (San Diego CA); rat anti-mouse CD16/CD32 Ab is from BD Pharmingen (Milan Italy); osmotic pump (Alzet model 1007D, 100 μl; 0.5 μl h^−1^), cannula (Azlet brain infusion kit 3) are from Charles River, Italy.

### Mice and cell lines

All the experiments were approved by the Italian Ministry of Health in accordance with the guidelines on the ethical use of animals from the European Community Council Directive of 24 November 1986 (86/609/EEC). We used *C57BL/6* mice and *CB17/Icr-Prkdcscid/IcrIcoCrI* (SCID) mice from Charles River Laboratories, and heterozygous *CX3CR1*^*+/*^GFP mice (Jackson Laboratories). We always used male mice, 3 weeks or 2 months of age.

The GL261 murine glioma cells (provided by Dr Serena Pellegatta, Mario Negri Institute Milan) and U87MG human glioma cells (ATCC) were cultured in DMEM supplemented with 20% or 10%, respectively, FBS.

### Environmental enrichment

Mice were housed for 5 weeks in EE: 10 mice for cage (36 cm × 54 cm × 19 cm), in the presence of an assortment of objects, including climbing ladders, seesaws, running wheel, balls, plastic and wood objects suspended from the ceiling, paper, cardboard boxes and nesting material. Toys were changed every 2 days, and the bedding every week. Control SE mice were in pair, in standard cages: 30 cm × 16 cm × 11 cm. Both EE and SE groups received chow and water *ad libitum*, on a 12-h light/dark cycle.

### Electrophysiological recordings

Mice were anaesthetized with halothane and killed. Whole brains were immersed in ice-cold artificial cerebrospinal fluid solution containing (in mM): NaCl 125, KCl 4.4, CaCl_2_ 2.5, MgSO_4_ 1.5, NaHPO_4_ 1, NaHCO_3_ 26 and glucose 10, oxygenated with 95% O_2_ and 5% CO_2_ (pH 7.4). Transverse hippocampal slices (350-μm thick) were cut with a vibratome (DSK, Japan), allowed to recover for 2 h and transferred to the interface slice-recording chamber (BSC1, Scientific System Design, at 30–32 °C and superfused at 1.5 ml min^−1^). A concentric bipolar stimulating electrode was positioned in the stratum radiatum (0.05 Hz) of the Schaffer collateral pathway. Orthodromically evoked fEPSPs were recorded with artificial cerebrospinal fluid-filled glass micropipette (0.5–1 MΩ) and averaged over 1 min (*n*=3). For LTP experiments, fEPSPs (35–45 min post induction) were normalized to baseline values (0–10 min) before LTP induction (HFS, 1 train, 100 Hz, 1 s). fEPSPs were recorded and filtered (1 kHz) with the Axopatch 200A amplifier (Axon Instruments, CA, USA) and digitized at 10 kHz with A/D converter (Digidata 1322A, Axon Instruments).

### Preparation of CD133+ GL261 cells

Single-cell suspensions from cultured GL261 were incubated for 10 min at 4 °C with rat anti-mouse CD16/CD32 Ab (1:250) and for 30 min at 4 °C with CD133 Ab (1:75). The CD133^+^-GL261 cell population was isolated using phycoerythrin anti-mouse CD133 Ab using a BD FACS AriaII (BD Biosciences). The purity of cell population was verified using flow cytometry (~90%). Sorted CD133^+^ GL261 cells were maintained in DMEM with 20 ng ml^−1^ fibroblast growth factor-2, 20 ng ml^−1^ EGF and Heparin 10 U ml^−1^, for 24 h before brain transplantation.

### Intracranial injection of glioma cells

After 5 weeks in EE or SE, mice were anaesthetized with chloralhydrate (400 mg kg^−1^, i.p.) and placed in a stereotaxic head frame. Animals were injected with 7.5 × 10^4^ GL261 or CD133^+^-GL261 cells, and 5 × 10^4^ U87MG cells: a median incision of ~1 cm was made, a burr hole was drilled in the skull and cells were injected in the right striatum (−2 mm lateral, +1 mm anteroposterior from the bregma). Cell suspension in PBS (4 μl) was injected at 1 μl min^−1^ and 3 mm depth. Mice were then again housed in EE or SE and analysed after 17 days. The 17-day period was chosen on the basis of tumour dimension and survival time.

### Histopathological evaluation of tumour volume and invasiveness

Seventeen days after GL261 injection, their brains were isolated and fixed in 4% buffered formaldehyde for morphological evaluation. Coronal brain sections (20 μm), prepared using the standard procedures, were stained with haematoxylin and eosin. A section every 100 μm was collected, and the tumour area was evaluated using Image Tool 3.00. For analysis of tumour invasiveness, glioma cells protruding more than 150 μm from the main tumour mass were counted in at least 20 fields, obtained from six slices per mice.

### Brain-clearing process

The brains were clarified using the 3Disco method^16^, dehydrated at room temperature in successive bathes of 50% THF overnight, 80% THF for 2 h to 100% THF twice for 1 h. The brains were incubated in 100% Di-ChloroMethan and then transferred overnight into the clearing medium (100% DiBenzylEther), changed once before sample observation.

### Three-dimensional imaging of cleared brains

Whole cleared brains of adult mice were imaged using a home-made light-sheet ultramacroscope designed as an open-source versatile ultramicroscopy solution^40^. The specimens were placed on the Z-stage of its optical/sample bench in a cubic glass cuvette filled with the clearing medium. The cuvette was illuminated sequentially by both sides with a 561-nm laser light sheet of 10 mW (LBX-4C, Oxxius, Lannion, France). The images were taken with a charge-coupled device camera (ORCA AG, Hamamatsu, France) through a 617/73 filter on a macroscope, equipped with a PlanApo × 1/0.25 or × 2/0.5 objective (MVX10, Olympus, France). The image acquisition piloted by the μManager^41^ software was synchronized with the z-stage movement (10 or 20 μm steps) to obtain three-dimensional image stacks of the sample.

### Brain and tumour volume measurements

The three-dimensional images were analysed using the ImageJ software (http://imagej.nih.gov/ij/, 1997–2012). Multiplying the surface measurement (after maximal projection of the image stack and thresholding or outlining the brain or tumour) by the height (number of slices including the brain or tumour multiplied by the z gap between slices) gave cylinder-like volume estimations of the brains and the tumours.

### Survival analysis

Following cell injection, mice were monitored daily. The end point was defined by the lack of physical activity or death. Survival was calculated using the Kaplan–Meier method, and statistical analysis was performed using a log-rank test.

### BrdU injection

Seventeen days after cell injection, BrdU was intraperitoneally (i.p.) injected (50 mg kg^−1^). Two hours later, mice were killed and their brains processed for immunostaining.

### Immunostaining

Cryostat brain sections (10 μm) were incubated with 3% goat serum in 0.3% Triton X-100 for 1 h at RT, and overnight at 4 °C with specific Abs in 1% goat serum, 0.1% Triton X-100 at the following dilutions: F4/80 (1:50), CD68 (1:200), CD45 (1:400), CD69 (1:200), NK1.1 (1:200), BrdU (1:200) and GFAP (1:1,000). Sections were then stained with fluorophore-conjugated secondary Abs and Hoechst for nuclei visualization. For BrdU staining, sections were incubated 15 min in 1 N HCl for 25 min in 2 N HCl and then neutralized in 0.1 M borate buffer. For F4/80 staining, coronal sections were first heated in 20 min in citrate buffer (pH 6.0) at 95–100 °C.

### Image acquisition and data analysis

Fuorescence images were digitized using a CoolSNAP camera (Photometrics) coupled to a ECLIPSE Ti-S microscope (Nikon) and processed using the MetaMorph 7.6.5.0 software (Molecular Device). Slices were scanned by consecutive fields of vision (× 10) to build a single image per section. Data were expressed as area occupied by fluorescent cells versus total tumour area (by converting pixel to mm^2^). For comparison between different treatments, at least 12 coronal sections per brain around the point of injection were analysed.

### Isolation of CD11b^+^ cells and extraction of total RNA

Brains of glioma-bearing mice were cut into small pieces and single-cell suspension was achieved by enzymatic digestion in trypsin (0.25 mg ml^−1^), in Hank’s balanced salt solution (HBSS) and mechanical dissociation using a wide-tipped pipette. Cell suspension was applied to a 30-μm cell strainer and immediately processed for MACS Micro Bead separation. The CD11b+ cells were magnetically labelled with CD11b Micro Beads. The cell suspension was loaded on a MACS Column (Miltenyi Biotec) placed in the magnetic field of a MACS Separator and a negative fraction was collected. After removing the magnetic field, CD11b^+^cells were eluted as positive fraction. Vitality and purity of CD11b^+^cells were assessed using flow cytometry (FACS). On sorting the positive and negative fractions, total RNA was isolated with the RNeasy Mini Kit and processed for RT–PCR. The quality and yield of RNAs were verified with Ultraspec2000 UV/Visible (Pharmacia Biotech).

### RT–PCR

Samples were lysed in Trizol reagent for isolation of RNA. Reverse transcription reaction was performed in a thermocycler using IScript TM RT Supermix (Bio-Rad) under the following conditions: incubation, 25 °C, 5 min; reverse transcription, 42 °C, 30 min; inactivation, 85 °C, 5 min. RT–PCR was carried out in a I-Cycler IQ Multicolor RT–PCR Detection System using SsoFast EvaGreen Supermix (Bio-Rad). The PCR protocol consisted of 40 cycles at 95 °C, 30 s and at 60 °C, 30 s. For quantitative analysis the comparative Threshold Cycle (*C*_t_) method was used, while normalizing to *C*_t_ value of GAPDH in the same sample. Relative quantification was performed using the 2^−ΔΔ*C*t^ method^42^ and expressed as fold changes in arbitrary values. Primers are listed in Supplementary Table 3.

### Plasmid construction

The piggy Bac transposon-based vector ePB-BSD-PGK-tagRFP was built by replacing the TRE promoter of the ePB-BSD-TRE-tagRFP construct^43^ with the constitutive human phosphoglycerate kinase (PGK) promoter.

### GL261 glioma cell transfection

Twenty-four hours before transfection, 50% of the GL261 growth medium was replaced with fresh culture medium. For transfection, 100 μl of DMEM was mixed with 4 μl of Magnetofection NeuroMag (OZ Bioscience, France) and 1.6 μg of the ePB-BSD-PGK-tagRFP vector and 0.4 μg of the piggy Bac transposase encoding plasmid. The mixture was incubated for 15–20 min at room temperature, and thereafter distributed dropwise over the culture. Cells were then placed on a magnetic plate and incubated for 15 min at 37 °C. Transfection was terminated by substitution of 50% of incubation solution with the fresh culture medium. Cells were selected for blasticidin resistance (10 μg ml^−1^).

### Measurement of BDNF

After 17 days from glioma transplantation, ipsilateral and controlateral hemispheres of EE or SE mice were disrupted with homogenizer and analysed for BDNF content using a sandwich ELISA.

### Intrastriatal BDNF and IL-15 infusion

Eight-week-old male *C57BL/6* mice were anaesthetized with chloralhydrate (400 mg kg^−1^, i.p.), placed in a stereotaxic head frame and injected in the right striatum (1 mm anteroposterior, 2 mm lateral and 3 mm deep, according to the atlas) with a Hamylton syringe. BDNF and IL-15 were dissolved in PBS and infused at the rate of 1 μl min^−1^: BDNF (100 ng ml^−1^ per 3 μl) was infused for 48 h before and during glioma transplantation; IL-15 (50 ng ml^−1^ per 3 μl) or vehicle were infused during and 10 days after glioma transplantation.

### BDNF and IL-15 delivery with micro-osmotic pumps

Ten days after glioma transplantation, mice were anaesthetized and implanted with an osmotic pump in the right striatum using the stereotaxic apparatus. A cannula was implanted through the same hole used for glioma transplantation and was sealed with dental cement before connecting to the pump. The pump was placed into a subcutaneous pocket in the dorsal region. The pumps were filled up with vehicle (PBS), BDNF (60 ng μl^−1^) or IL-15 (30 ng μl^−1^). Before surgery, the pumps and the tubes were incubated at 37 °C overnight in a sterile saline. The experiment continued for 7 days after the pump implantation. BDNF and IL-15 doses were selected on the basis of the *in vitro* experiments.

### Leukocyte isolation and FACS analysis

Seventeen days after the glioma transplantation, mice were intracardially perfused with PBS, brain rapidly removed, hemispheres separated and placed into 5 ml of ice-cold PBS containing 0.2% bovine serum albumin, 0.01 M EDTA and 1 mg ml^−1^ deoxyribonuclease I. The hemispheres were disrupted in a glass homogenizer and passed through a 70-μm nylon cell strainer. The suspension was centrifuged at 400*g* for 10 min at room temperature; the pellet was resuspended in 4 ml of 30% Percoll and overlaid on the top of a gradient containing 3.5 ml of 37% and 3.5 ml of 70% Percoll solution. Percoll was prepared by diluting in HBSS. The gradient was centrifuged at 900*g* for 20 min at room temperature; cells were collected from the 37 to 70% interface (~3 ml) and washed once with HBSS containing 10% FBS. Blood samples were collected into heparin-containing tubes by tail vein puncture. After removal of contaminating erythrocytes, cells were counted, and 30–50 μl of whole blood cells were washed and resuspended in staining buffer. Cells were then blocked by incubation in ice with anti-CD16/CD32 (24G2) to prevent nonspecific and Fc-mediated binding. After 10 min, a mixture of the appropriate antibodies was added. Cells were further incubated on ice for 30 min, washed and analysed using a FACSCanto II (BD Biosciences). Data were elaborated using the FlowJo Version 7.6 software (Tree Star).

### NK1.1^+^ cell depletion

Depletion was performed using a blocking Ab against NK1.1, which recognizes an epitope of the NKR1Pc-activating receptor (PK136). C57BL/6 mice (*n*=5 per group, EE or SE) were i.p. injected with 200 μg (in 100 μl) of anti-NK1.1 Ab 2 days before, during and 2 and 7 days after glioma transplantation. Seven and seventeen days after glioma surgery, NK cell depletion from the blood sample was monitored with FACS. Tumour size were measured on day 17. For survival analysis, NK1.1 Ab injection was repeated once a week until the end point.

### Microglia cultures

Microglia cultures were obtained from mixed glia cultures derived from the cerebral cortices of postnatal day 0–1 (p0–p1) C57BL/6 mice. Cortices were chopped and digested in 15 U ml^−1^ papain for 20 min at 37 °C. Cells (5 × 10^5^ cells cm^−2^) were plated on flasks coated with poly-L-lysine (100 mg ml^−1^) in DMEM supplemented with 10% FBS, 100 U ml^−1^ penicillin and 0.1 mg ml^−1^ streptomycin. After 7–9 days, cells were shaken for 2 h at 37 °C to detach and collect microglial cells. These procedures gave almost pure microglial cell populations.

### Proliferation assay *in vitro*

GL261, plated at a density of 13 × 10^4 ^cm^−2^, were treated with BDNF and/or EGF (both at 100 ng ml^−1^) for 24 or 48 h. Cell number was measured treating cells with detergent containing buffer (0.05% ethyl hexadecyldimethylammonium bromide, 0.028% acetic acid, 0.05% Triton X-100, 0.3 mM NaCl, 0.2 mM MgCl_2_ in PBS pH 7.4) and counting them in a hemacytometer as described^44^. In all figures, results are expressed as % of cell proliferation, taking as 100% the untreated cells in control conditions.

### Precipitation assay

For RhoA pull-down assay, 24 h before stimulation, GL261 were seeded (3 × 10^6^ cells) with 2.5% FBS, and 2 h before treatment, starved in DMEM. Subsequently, GL261 were exposed for 5 min to BDNF (100 ng ml^−1^) and/or EGF (100 ng ml^−1^). Afterwards, cells were lysed in buffer containing 50 mM Tris/HCl, 150 mM NaCl, 1% Triton X-100, 0.15% Na deoxycholate, 0.1% SDS, 50 mM Napyruvate 100 mM NaF, 1 mM EDTA, 1 mM EGTA, 10 mM MgCl_2_, 1 mM phenylmethyl sulphonyl fluoride, 10 μg ml^−1^ leupeptin and 20 μg ml^−1^ aprotinin, Rhotekin beads (Millipore 23 μl) were added to the lysates and incubated at 4 °C for 3 h. The beads were then washed with the lysis buffer and the result pellets were mixed with 2 μl dithiothreitol and 25 μl SDS sample buffer, boiled 5 min and analysed using western blot analysis.

### Western blot

For protein extraction, GL261 were stimulated as previously described. The same amount of proteins (20 or 50 μg per sample) was loaded on SDS polyacrylamide gel and electrophoretically transferred to a nitrocellulose paper. Blots were incubated with specific primary and horseradish peroxidase-conjugated secondary Abs, and immunoreactivity detected by enhanced chemiluminescence. Densitometric analysis was carried out with Chemi-Doc (Bio-Rad). Images have been cropped for presentation and full-size images are shown in Supplementary Fig. 6

### Phagocytosis

Microglial cells were co-cultured with GL261 as described, in the presence or in the absence of BDNF (100 ng ml^−1^) for additional 24 h. LPS (500 ng ml^−1^) was used as the positive control. Red fluorescent FluoSpheres (0.05% or 1.8 × 10^7^ spheres ml^−1^) were added for 1 h in 0.1% BSA, and nuclei were stained with Hoechst. Cells were deeply washed and fixed in 4% paraformaldehyde for 15 min. Phagocytizing cells (scoring as positive only cells with at least three FluoSpheres to avoid possible false-positives because of sphere adhesion to the cell surface) were counted in at least 20 random fields (594 nm, Axioscope2; Carl Zeiss, Oberkochen, Germany).

### Boyden chamber chemotaxis assays

Glioma cells were preincubated in the chemotaxis medium (DMEM without glutamine, 100 IU ml^−1^ penicillin G, 100 μg ml^−1^ streptomycin, 0.1% BSA and 25 mM HEPES, pH 7.4) supplemented with AraC 5 μm, for 15 min, and plated (4 × 10^4^ cells) on poly-L-lysine-coated transwells (8 μm pore size) in this same medium. The lower chamber contained BDNF (100 ng ml^−1^), EGF (100 ng ml^−1^) or vehicle. After 18 h at 37 °C, cells were treated with ice-cold 10% trichloroacetic acid, cells adhering to the lower side of the filter were stained (50% isopropanol, 1% formic acid and 0.5% (wt/vol) brilliant blue R250) and counted (20 fields, × 40 objective). For microglia (3 × 10^4^ cells), the medium alone or conditioned 24 h by GL261 cells in presence or absence of BDNF (100 ng ml^−1^) was used as chemoattractant (3 h).

### General criteria adopted for the experiments

For each experiment, the sample size (*n*) was chosen considering the following relation: *n*≥2sigma (Zalpha/D)2, where sigma is substituted by an estimate of variance (s2); alpha is at 0.05 (and Zalpha=−2) and D is the difference among treatments. Criteria of animal exclusion/inclusion were pre-established; animals considered for the analysis were selected for age. From some analyses we excluded mice that after glioma transplantation did not develop cerebral tumour. At weaning, pups from different colonies were mixed together and mice were randomly allocated to SE or EE. The investigators performing the different analyses always received the samples from a third laboratory member, who was not involved in that specific experiment, to ensure blinding to the group allocation.

### Statistical data analysis

Data shown are the mean±s.e.m. Where appropriate, Student’s *t*-test or ANOVA was used, as detailed. Statistical analyses were conducted the using Sigma Plot 11.0 software.

## Author contribution

S.G. planned and performed all the experiments; G.D.A. performed experiments of tumour transplantation; G.C. performed experiment with micro-osmotic pumps; F.B. performed experiments of brain-clearing; L.M. performed electrophysiology experiments; A.R. performed the transposon-based vector experiments; A.P. and F.M. performed the FACS and pull-down assays; V.E. contributed to experiment planning; C.La performed PCR experiments; G.Ben. and G.Ber. perfomed FACS analyses; A.S. contributed to experiment planning and paper writing; C.Li. supervised and planned the experiments and wrote the paper. All authors contributed to article preparation.

## Additional information

**How to cite this article:** Garofalo, S. *et al*. Enriched environment reduces glioma growth through immune and non-immune mechanisms in mice. *Nat. Commun.* 6:6623 doi: 10.1038/ncomms7623 (2015).

## Supplementary Material

Supplementary Information Supplemetary figures

Supplementary Movie 1 *z* stacks of cleared brains from a SE housed mice injected with tagRFP-GL261 cells and analyzed after 17 days. In movie (1 X magnification) the gap between images is 20μm, size of the box around the tumor = 2x2mm.

Supplementary Movie 2 *z* stacks of cleared brains from a EE housed mice injected with tagRFP-GL261 cells and analyzed after 17 days. In movie (1 X magnification) the gap between images is 20μm, size of the box around the tumor = 2x2mm.

Supplementary Movie 3 *z* stacks of cleared brains from a SE housed mice injected with tagRFP-GL261 cells and analyzed after 17 days. In movie (2 X magnification) the gap between images is 5μm.

Supplementary Movie 4 *z* stacks of cleared brains from a EE housed mice injected with tagRFP-GL261 cells and analyzed after 17 days. In movie (2.5 X magnification) the gap between images is 5μm.

## Figures and Tables

**Table 1:** Expression of selected genes in contra- (C) and ipsilateral (I) hemispheres of glioma-bearing mice housed in SE or EE.

	**SE**		**EE**	
	**C**	**I**	**C**	**I**
*bdnf*	1.00±0.01	0.75±0.15	1.96±0.30*	2.24±0.29*
*il-15*	1.00±0.05	0.83±0.07	1.44±0.13*	1.63±0.06*
*ccl2*	1.00±0.01	50.55±10*	0.87±0.10	9.04±0.72*
*cxcl10*	1.00±0.04	5.01±0.36**	0.06±0.01*	1.62±0.16*
*il-6*	1.00±0.01	3.06±0.01*	1.28±0.16	1.13±0.05*

EE, enriched environment; RT–PCR, real-time–PCR; SE, standard environment.

Results of RT–PCR analysis are shown as fold increases versus C of mice housed in SE for each condition (*n*=4 Student’s *t*-test; *P<0.05, **P<0.01).

**Table 2:** Expression of selected genes in CD11b^+^ and CD11b^−^ cells isolated from contra- and ipsilateral hemispheres of glioma-bearing mice housed in SE or EE.

	**CD11b**^**+**^				**CD11b**^**−**^			
	**SE**		**EE**		**SE**		**EE**	
	**C**	**I**	**C**	**I**	**C**	**I**	**C**	**I**
*bdnf*	1.00±0.01	0.80±0.06	2.06±0.20**	2.18±0.26*	1.00±0.01	0.91±0.10	1.86±0.16**	1.80±0.06**
*il-15*	1.00±0.01	1.20±0.13	2.15±0.41*	2.14±0.40*	1.00±0.02	1.06±0.02	1.07±0.02	0.76±0.10

EE, enriched environment; RT–PCR, real-time–PCR; SE, standard environment.

Results of RT–PCR analysis are shown as fold increases (versus C) of mice housed in SE (*n*=3–4 Student’s *t*-test; *P<0.05; **P<0.01).
