# Supplementary material for: Enriched environment reduces glioma growth through immune and non-immune mechanisms in mice
Source: Nat Commun. 2015 Mar 30;6:6623. doi: 10.1038/ncomms7623 (PMC4389244; doi:10.1038/ncomms7623)
Supplement: Supplementary Information — Supplemetary figures [file ncomms7623-s1.pdf]

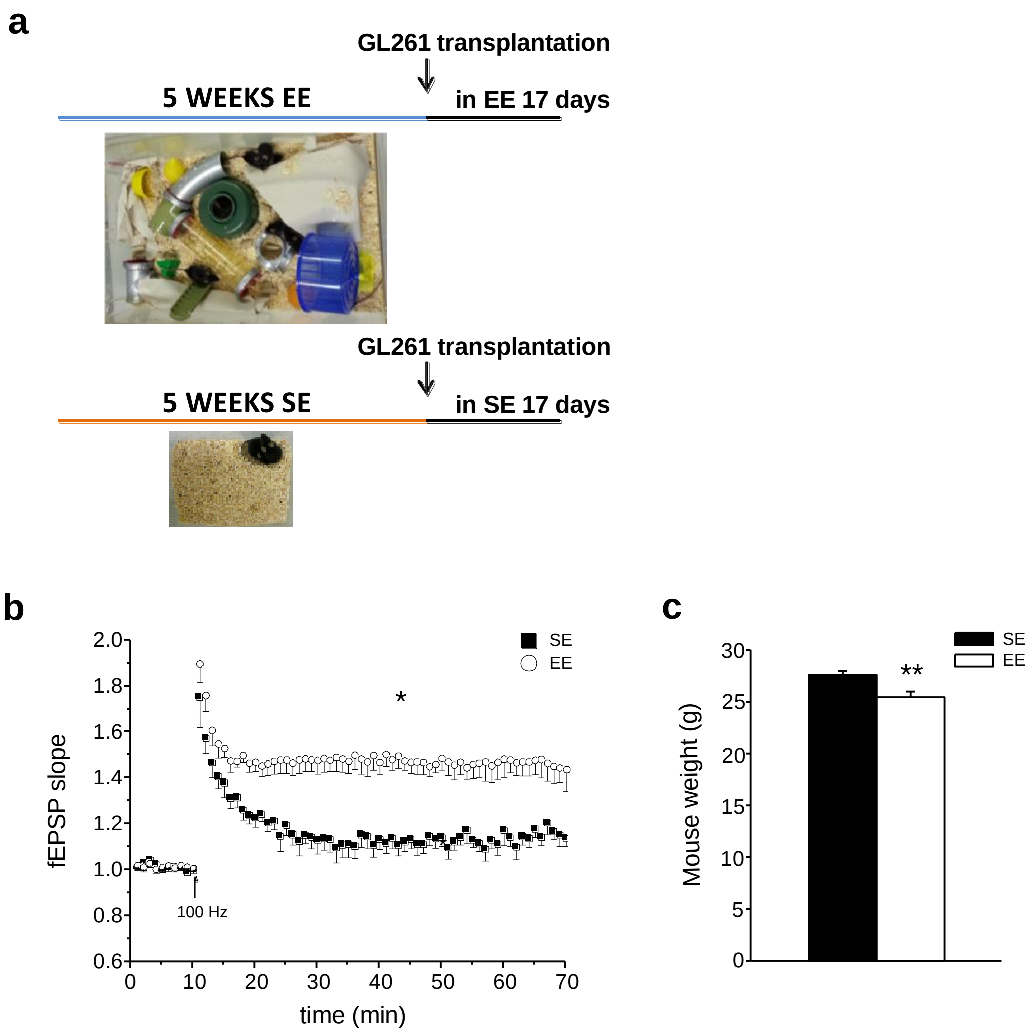

Supplementary Figure 1 ***Housing in EE improves hippocampal plasticity and reduces mice weight***. (**a**) Experimental plan showing the different housing conditions and the timing of glioma transplantation in mice. (**b**) Field excitatory post-synaptic potential (fEPSP) recorded from the CA1 region of hippocampus of SE or EE mice. Points represent averages (± SEM) of normalized fEPSP slopes evoked every 20 sec. Arrow indicates the time of application of high frequency stimulation (HFS, 100 Hz train). Note the HFS induced LTP of fEPSP slope only in EE (open circle) not in SE mice (dark square, n=6, 3 experiments ,* $p<0.05$). (**c**) Graph bars indicate the mean mouse weight (± SEM) upon SE or EE housing for 5 weeks, as indicated. ** $p<0.01$ Student's *t*-test n=12 mice per condition.

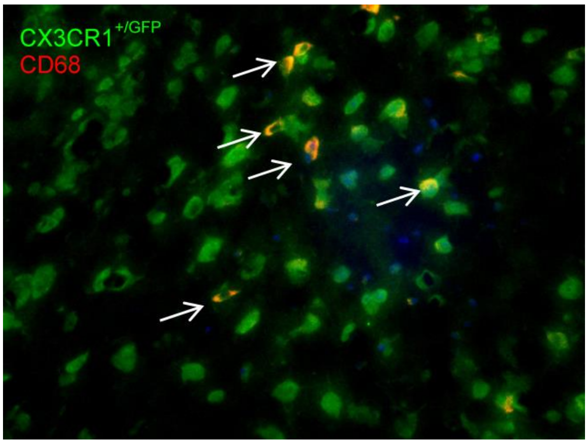

Supplementary Figure 2 ***Expression of CD68 only on GFP+ cell***. Representative immunofluorescence of CD68+ cell (red) co-labeled with GFP+ cell (green).

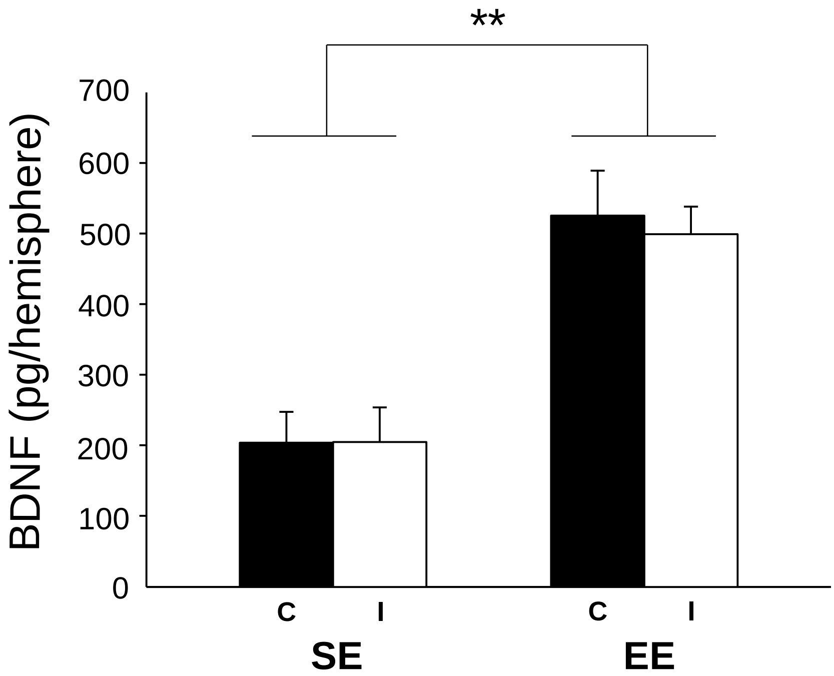

Supplementary Figure 3 ***BDNF expression increases in the brain of EE mice.*** BDNF protein level was measured by ELISA in contralateral (C) and ipsilateral (I) cerebral hemispheres of glioma bearing SE or EE mice. Results are expressed as pg of BDNF per hemisphere. ** $p<0.01$ one-way ANOVA n=3 mice per condition.

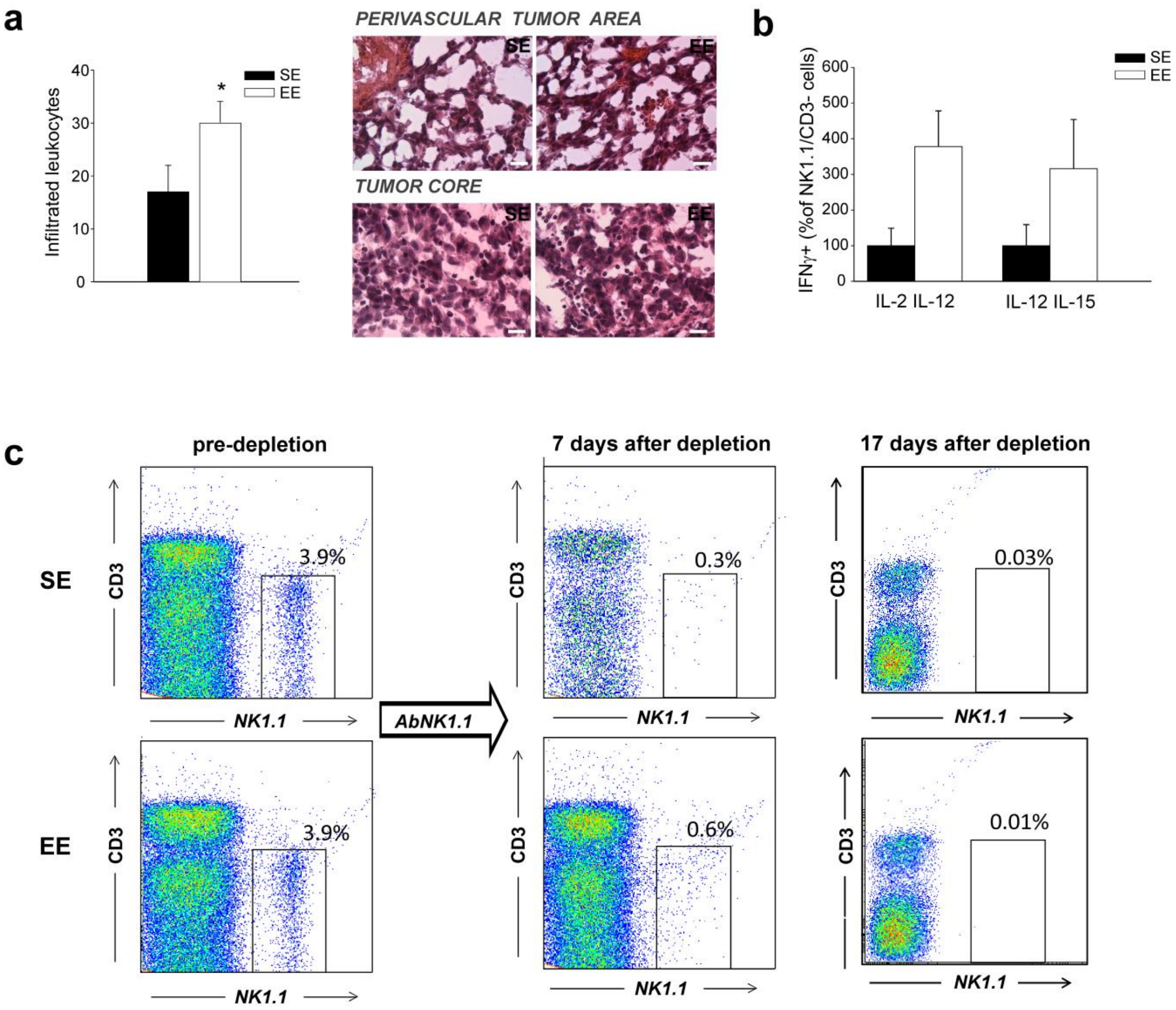

Supplementary Figure 4 *NK cell infiltration in glioma upon housing in SE and EE*. (a) Coronal brain sections from SE or EE mice were stained with hematoxylin/eosin and analyzed for leukocyte infiltration 17 days after glioma transplantation, as indicated. Data are expressed as number of infiltrated lymphocytes in tumor area (± SEM) (scale bars: 100 μm; * $p<0.05$ Student's *t*-test, n=4 mice per condition). Representative stained slices are shown on the right. (b) Percentage of IFNγ$^+$ cells in the CD3$^-$/NK1.1$^+$ cell population obtained from the blood of SE or EE mice after overnight stimulation with IL-2 (100U/ml)//IL-12 (100ng/ml) or IL-12 (100ng/ml)/IL-15 (50ng/ml) (n=2). (c) FACS analysis of CD3$^-$/NK1.1$^+$ cells in plasma of mice before and after treatment with NK1.1 Ab (i.p.) to deplete NK cells, as indicated. Panels are representative of at least five mice per condition.

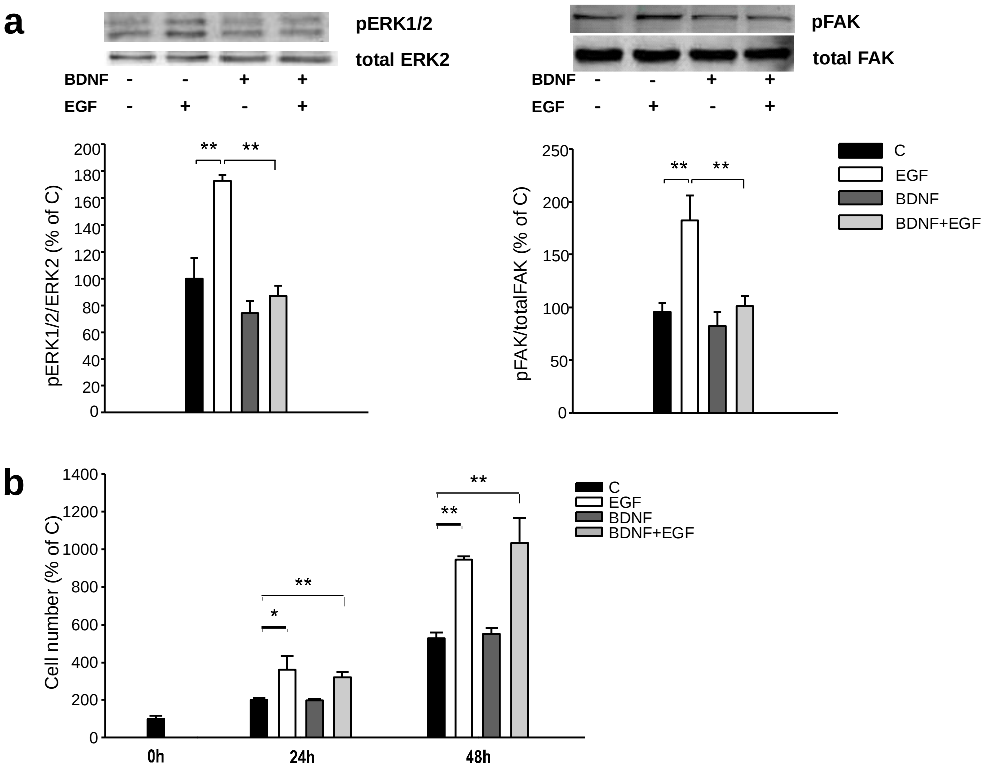

Supplementary Figure 4 ***Effects of BDNF on GL261 cells.*** (a) BDNF (100 ng/ml, 5 min) inhibits EGF (100 ng/ml, 5 min) induced ERK1/2 (left) and FAK (right) phosphorylation in GL261 cells. Data are shown as pERK1/2/ERK2 (left) and pFAK/FAK (right) and expressed as percentage of controls (C, n=4, ** p<0.01 one-way ANOVA). Representative western blots are shown on top of each bar graph. (**b**) GL261 cell count upon BDNF or EGF (both at 100 ng/ml) treatment for the indicated time points. Data are expressed as percentage of untreated cells (C) at time 0 (n=3).

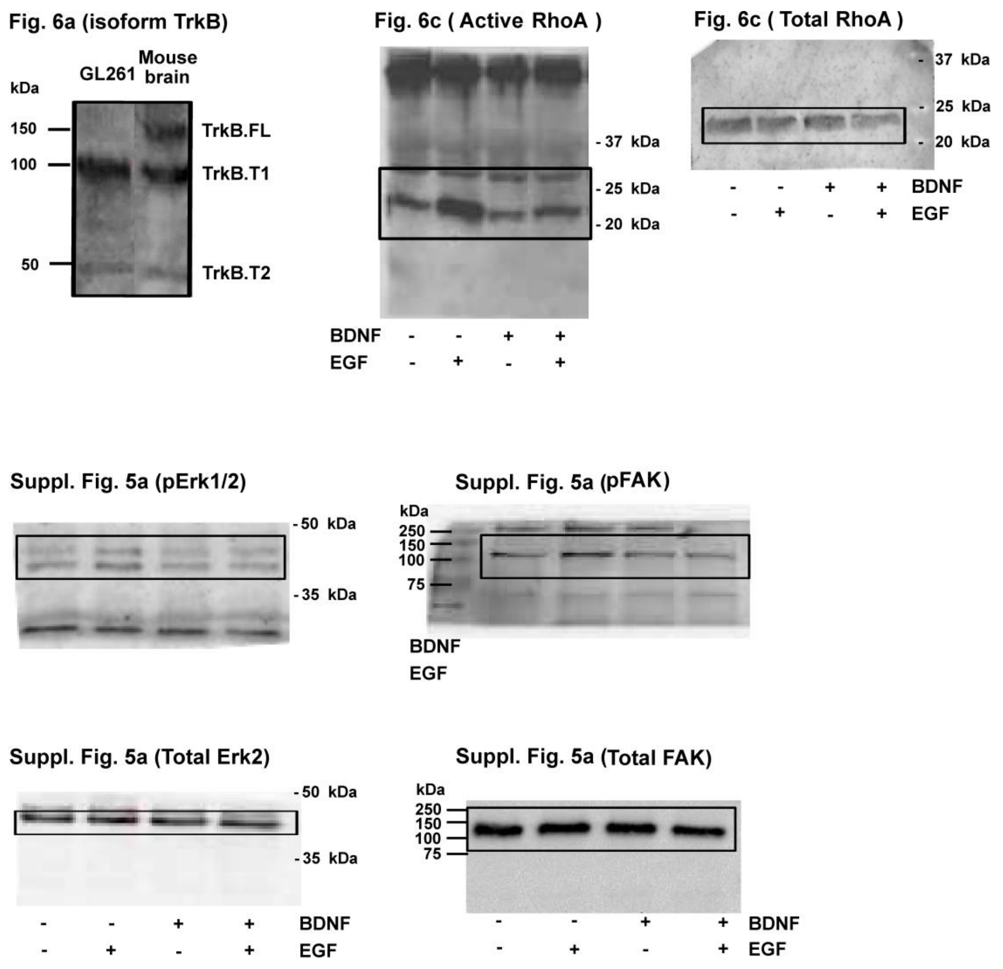

Supplementary Figure 5 Full blot of the indicated figures.

Supplementary Table 1 ***Expression of selected genes in contra- (C) and ipsi-lateral (I) hemispheres of sham operated mice housed in SE or EE.***

	SE		EE	
	C	I	C	I
**bdnf**	1.00±0.03	**1.21±0.06***	**1.83±0.22***	**2.28±0.26***
**il-15**	1.00±0.01	0.87±0.24	0.64±0.04	0.61±0.10
**ccl2**	1.00±0.04	0.97±0.10	0.98±0.12	0.93±0.06
**cxcl10**	1.00±0.02	0.73±0.24	**0.41±0.14***	**0.39±0.26***
**il-6**	1.00±0.01	0.88±0.18	1.18±0.35	0.96±0.14

Results of RT-PCR analysis are shown as fold increases *vs* C of mice housed in SE for each condition (n=3 Student *t*-test * $p<0.05$).

Supplementary Table 2 ***Expression of genes*** **for NK cell activating cytokines in** ***contra*** **- (C) and ipsi-lateral (I) hemispheres of glioma** ***bearing*** **mice housed in SE or EE.**

	SE		EE	
	C	I	C	I
**il-2**	1.00±0.09	**10.3±2.80***	**0.64±0.03***	**39.55±8.09****
**il-18**	1.00±0.05	1.27±0.35	0.73±0.05	1.00±0.14
**il-12p35**	1.00±0.08	1.10±0.22	0.82±0.11	1.16±0.18
**il-12p40**	1.00±0.05	0.95±0.04	0.98±0.19	1.02±0.22

Results of RT-PCR analysis are shown as fold increases *vs* C of mice housed in SE for each condition (n=4 Student *t*-test * $p<0.05$ ** $p<0.01$).

Genes	Forward	Reverse
il-15	CATCCATCTCGTGCTACTTGTGTT	CATCTATCCAGTTGGCCTCTGTTT
bdnf	TGAGTCTCCAGGACAGCAAA	TGTCCGTGGACGTTTACTTCT
cxcl10	AAGTGCTGCCGTCATTTTCT	CTTCCCTATGGCCCTCATTC
il-6	GATGGATGCTACCAAACTGGA	TCTGAAGGACTCTGGCTTTG
ccl2	AGGTCCCCTGTCATGCTTCTG	TCTCCAGCCTACTCATTGGG
il-2	CCTGACAGGATGGAGAATTACA	TCCAGAACATGCCGCAGAG
il-18	GCCTCAAACCTTCCAAATCA	TGGATCCATTTCCTCAAAGG
il-12p35	CTCCTGGACCACCTCAGTTTG	CGGTCATCTGCCGCAAA
il-12p40	GGTGAAGGCATGGGAACATT	TGCCCATTCGCTCCAAGA
gapdh	TCGTCCCGTAGACAAAATGG	TTGAGGTCAATGAAGGGGTC
tk+(603 bp)	TCAGCAACGACGATGACTCT	AGTGTTGGGATGCCAGGTAG
t1 (360 bp)	ACTGACATCGGGGATACTAC	GTGTTCTTCTGCTGCTTCTC
t2 (232 bp)	CTGTTGCCTATCCCAGGAAG	GAGAGGCACAATCCAATGAG
h-□-actin	TAAGGAGGAGCTGTGCTACG	GGAGCAATGATCTTGATCTTC

Supplementary table 3 PCR primer sequences
